# Twenty-Eight-Day-Long Delirium Tremens

**DOI:** 10.1177/2324709619847228

**Published:** 2019-05-03

**Authors:** Paritosh Kafle, Amrendra Kumar Mandal, Binav Shrestha, Bikash Bhattrai, Manjul Bhandari, Shambhu Bhagat, Bijoy Shankar Kar, Dikshya Sharma, Vijay Gayam

**Affiliations:** 1 Interfaith Medical Center, Brooklyn, NY, USA; 2 Maimonides Medical Center, Brooklyn, NY, USA

**Keywords:** delirium tremens, DT, benzodiazepines, phenobarbitone, midazolam, prolonged delirium tremens

## Abstract

Refractory alcohol withdrawal delirium is uncommon in day-to-day clinical practice. This case report presents a rare case of delirium tremens of unusually long duration that was complicated by the difficulty in tapering down benzodiazepines despite adding midazolam drip as well as phenobarbitone to the management regimen and excluding other possible diagnoses.

## Introduction

Two percent to 5% of hospitalized alcoholic patients are estimated to suffer from delirium tremens (DT), which has a mortality rate of about 1% to 15%.^1^ DT presents commonly on the third to fifth day after alcohol abstinence and lasts about 24 hours to 6 days, but in the rare instance the condition can last for weeks.^2-4^ In this article, we present a case where multiple groups, remarkable doses of medications including benzodiazepines, and exceptionally more extended treatment during 4 weeks were required to stabilize DT. A few decades earlier, the mortality related to DTs was estimated to be as high as 15%, but with improvements in understanding and care it now has decreased significantly.^5^ More recent estimates range from 2% to 5%.^3,6^

## Case Presentation

**Figure 1.**
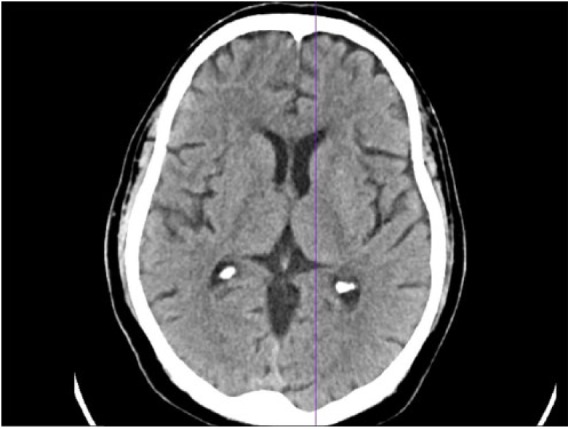
Computed tomography scan of the head showing normal imaging.

A 48-year-old male from Veteran shelter with a history of hypertension, alcohol dependence, and alcohol withdrawal seizures presented to the emergency department for worsening tremors and inability to walk well due to severe shakiness and unsteady gait for 1 week. On admission, he reported feeling anxious, agitated, and excessive sweating but denied nausea, vomiting, headache, auditory, visual, or tactile hallucinations. Review of the system was otherwise negative. He was not taking any medication and denied smoking or using illicit drug use. He endorsed drinking around 4 cans of 24 ounces of liquor daily and had been doing so for the last 20 years, with his last drink being on the day of admission. He had multiple detox admissions at our facility, with the last one being 6 months prior to the admission. Physical examination revealed an anxious-looking white Hispanic male with mild diaphoresis, tachycardia, tachypnea, and tremulousness. Computed tomography scan of the head on admission did not reveal any intracranial pathology. We excluded alcohol-related dementia and hepatic encephalopathy, based on his mentation, Mini-Mental State Examination, mild ammonia elevation, ultrasonographic evidence of mild hepatic steatosis without any increased nodularity of the liver, and a viral serology that was negative for hepatitis panel including hepatitis A, hepatitis B, and hepatitis C. The patient got admitted to step-down unit and intensive care unit (ICU) for severe alcohol withdrawal and was managed with lorazepam tapering as per hospital protocol. Along with fluid resuscitation, thiamine and folic acid were supplemented. The first 2 days of the hospital stay were unremarkable; however, on the third day, his condition deteriorated. He gradually started to get confused, and by night, he was agitated and grossly disoriented while he was being treated with scheduled intravenous (IV) lorazepam at a dose 2 mg every 6 hours along with as-needed doses. Clinical deterioration warranted upgraded to ICU for DTs for close monitoring on the same day. Despite the ICU standards of care, the patient clinically worsened with marked confusion, complete disorientation, agitation, aggressive behavior, and intense tactile hallucinations—the hallmarks of DTs despite being on escalating doses of IV lorazepam. Regular laboratory draws did not reveal any metabolic abnormalities. Neurology team was on board who suggested the possibility of Wernicke’s encephalopathy despite the patient being on IV thiamine. Other differentials included alcohol-induced dementia/amnestic syndrome; however, our treatment continued along the lines of alcohol withdrawal. With every passing day, the dose of benzodiazepines escalated, and lorazepam was subsequently replaced with continuous IV infusion of midazolam on the 9th day. He showed some improvement over the next 5 days and midazolam infusion was transitioned to lorazepam every 2 hours. However, on the 16th day of admission, he once again started manifesting the classic sign of DTs. Lorazepam dosing escalated again, and on the same day, he had to be restarted on midazolam infusion as he was not improving despite being on 2 mg of lorazepam every 15 minutes for an hour. The neurology team suggested other possibilities as the source of his confusion. There were no signs of infection or any other cause of worsening delirium. Strong history of drinking and classic presentation of DTs in the absence of any obvious metabolic, organic, or infective causes with normal complete blood count, complete metabolic panel, computed axial tomography scan of the head (Figure 1), and a negative lumbar puncture/cerebrospinal fluid analysis.

We reconsidered to continue the treatment of DTs and restarted him on midazolam IV infusion, which plateaued his symptoms, but he worsened with multiple attempts to taper midazolam. Given the circumstances, on the 24th day of his admission, we added phenobarbitone to the mix, starting at 60 mg every 8 hours while continuing midazolam infusion. Within 2 days of starting this new combination, we were able to taper off both the medications, and he was effectively transitioned to intermittent lorazepam infusion. Any attempts to escalate tapering were met with new symptoms of severe alcohol withdrawal. We subsequently were able to discontinue lorazepam following 28 days of minimal tapering. Significant improvement of his symptoms with eventual discharge to an inpatient drug rehabilitation program. He completed his inpatient drug rehabilitation of 28 days and was devoid of any alcohol withdrawal symptoms throughout his stay, before relapsing back to drinking again following discharge.

## Discussion

Our case is unique in a way that unlike other published case reports of persistent DTs there was no contributing cause leading to prolonged and refractory DTs. Prolonged DT was diagnosed after excluding all other possible diagnoses mimicking DTs including complete toxicology screen. Alcoholics are at higher risk of abusing additional substances especially sedative-hypnotics or anxiolytics, which is known to alter the course of withdrawal symptoms significantly. Our patient received a very high dose of lorazepam including midazolam and eventually was treated with phenobarbitone for the control of refractory DTs. The requirement of high-dose benzodiazepines in refractory DT can be theoretically explained by low in situ levels of gamma-amino butyric acid (GABA) or GABA receptor’s conformational changes acquired probably with prolonged heavy consumption of alcohol.^7,8^ Refractory DT has not been clearly defined in the medical literature, but DT is generally considered refractory if withdrawal control is not achieved despite administration of diazepam (50 mg) or lorazepam (10 mg) in the first hour or diazepam (>200 mg) or lorazepam (>40 mg) in first 3 to 4 hours of management.^8^ Explanation of the higher benzodiazepine demand is not yet completely understood, and it has intricate, complex mechanisms. Resistance to high-dose benzodiazepine is presumed to be a central effect as the pharmacokinetics of benzodiazepines (diazepam) is not shown to be altered in alcohol withdrawal.^9^ Benzodiazepines group of drugs are inhibitory neurotransmitters and exerts its effect on central nervous system GABA receptors, and so does alcohol.^10,11^ Minimal reports have been found in the literature of persistent DT for weeks and its management. Feuerlein and Reiser found that among almost 800 cases of DT, 62% resolved within 5 days, whereas 6% persisted for 10 days or more.^12^ Patients experiencing protracted withdrawal delirium had multiple comorbid medical or surgical conditions in that study. Our report had only hypertension and no other comorbid condition.

Heavy alcohol consumption is known as the risk for prolonged delirium after cessation, which is also found in our report.^13^ We used short-acting benzodiazepine (lorazepam) to limit the complicating factors seen with longer acting benzodiazepines. Miller et al reported a case with 2 episodes of protracted alcohol withdrawal delirium.^4^ The first episode lasted approximately 6 weeks but was complicated by neurosurgical problems and 3 weeks long, with second episode occurring almost a year later. The occurrence of prolonged delirium may be higher in patients with complex comorbidities such as dementia or head injury, as seen in the later patient described.

## Conclusion

Delirium tremens are a common diagnosis in clinical practice lasting about a week, but the physician should keep in mind the diagnosis of persistent DT, which might require different classes of medications. Prolonged DT can lead to a diagnostic dilemma. A further higher level of studies is required to define the management and complications of prolonged DT.
